# Synovial chondromatosis combine with synovial tuberculosis of knee joint: a case report

**DOI:** 10.1186/s12887-021-03085-1

**Published:** 2022-01-03

**Authors:** Nan Zhou, Ke Fang, Djandan Tadum Arthur V, Runbin Yi, Feng Xiang, Jie Wen, Sheng Xiao

**Affiliations:** Department of Pediatric Orthopedics, Hunan Provincial People’s Hospital, the First Affiliated Hospital of Hunan Normal University, Changsha, 410005 Hunan China

**Keywords:** Synovial chondromatosis; synovial tuberculosis; pediatric knee

## Abstract

**Backgroud:**

Synovial chondromatosis is a rare synovial-derived metaplasia disease that comes from the formation of cartilage nodules within the synovial connective tissue of the joint. Knee tuberculosis is a disease caused mostly by the pulmonary tuberculosis and a few by tuberculosis of the digestive tract and lymphatic.

tube.

**Case presentation:**

Herein we report a 3-year-old child admitted by intermittent swelling of left knee joint with lameness for half a year, the patient received surgical treatment. The loose bodies filled in the joint cavity was taken out and the degenerative synovium was excised. Biopsy confirmed as synovial chondromatosis combined with synovial tuberculosis of knee joint. After 6 months follow-ups, knee swelling and claudication get totally recovered and the gait of patient recover back to normal.

**Conclusion:**

Careful investigation of children with knee pain is recommended to avoid misdiagnosis, Synovial chondromatosis combine with tuberculosis should be considered a differential diagnosis in a child with knee pain.

## Article summary

Herein we report a 3-year-old child confirmed as synovial chondromatosis combined with synovial tuberculosis of knee joint who received surgery and anti-tuberculosis treatment.

## Backgroud

Synovial chondromatosis (SC) is a benign lesion that can occur in any synovial joint, with an incidence of one in 100,000 And the male-to-female ratio is approximately 1.8:1, with adult males predominating [1]. The main features of SC are synovial hyperplasia and the formation of cartilage vesicles from connective tissue cells [2]. The differential diagnosis includes pigmented villonodular synovitis, secondary synovial chondromatosis, rheumatoid or other seronegative arthritis, septic arthritis including granulomatous infection, synovial hemangioma, synovial chondrosarcoma.

Synovial tuberculosis has a slow onset and mild symptoms [3]. It is more common in children and young adults with single joint. Synovial tuberculosis in double joints, multiple joints or extra osseous tuberculosis are extremely rare [4].

Herein we report a 3-year-old child confirmed as synovial chondromatosis combined with synovial tuberculosis of knee joint. There was no literature in English that ever reported synovial chondromatosis combined with the synovial tuberculosis.

## Case presentation

**Fig. 1:**
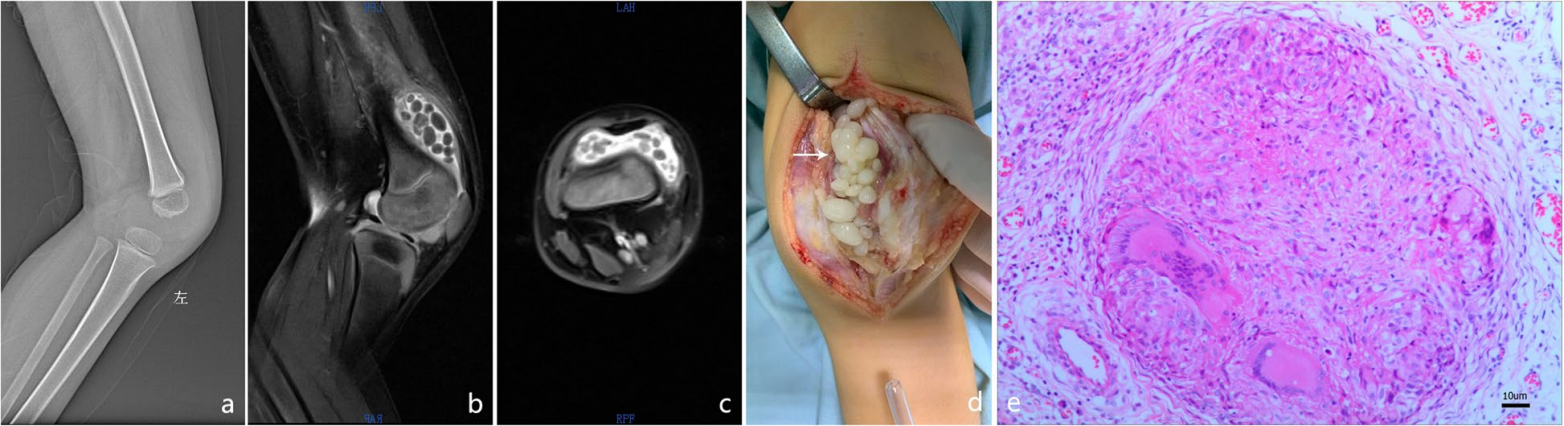
Radiologic, intra-operation and pathology result of left knee joint. **a** X-ray shows the change of the left knee joint space. **b**, **c** MRI revealed abnormal signal changes in the left knee joint. **d** There were a large number of white translucent round loose bodies (white arrow) overflowing in the joint cavity. **e** Pathological results shows granulomatous tissue, epithelioid cells and typical Langhans giant cell (black arrow)

The patient is a 3 year old male who experienced intermittent swelling of left knee joint with limp for half a year. The patient had low fever in the afternoon, night sweats, and crying at night. The parents also complained that the patient had significant weight loss in the past 6 months. There is no similar cases in patient’s family. Laboratory test: Blood Regular Test: White Blood Cells WBC:9.98*10^9^/L, Erythrocyte Sedimentation Rate (ESR): 15 mm/h, C-reactive protein (CRP): (−), Procalcitonin(−), T-SPOT(−), Anti-tuberculosis antibody (−),Tuberculin purified protein derivative (TPPD): (−), Chest X-ray(−). Ultrasound showed effusion in the suprapatellar capsule of the left knee, X-ray showed the change of the space of the left knee joint (Fig. 1a), Magnetic Resonance Imaging (MRI) showed abnormal signal focus in the suprapatellar capsule and the knee joint cavity, a small amount of effusion in the left knee joint cavity, and multiple nodules in the left popliteal fossa (Fig. 1b, c).

After been carefully accessment, the patient received surgical treatment (Fig. 1d). The operation was performed in supine position via anteromedial approach. After knee joint capsule open, a large number of loose bodies overflowed from the suprapatellar capsule and joint cavity. The synovium in the joint cavity was purplish red. The joint cavity was filled with loose bodies and was taken out The degenerative synovium was excised. The size of the loose body was different: (0.3 cm × 0.3 cm × 0.2 cm ~ 2.0 cm × 1.5 cm × 1.0 cm) it is white or milky white, the surface is smooth and translucent (Fig. 1d), the texture is tough, cartilage like and elastic, a few loose bodies are connected with the synovium in pedicle shape, and solid changes can be seen inside the loose body. Postoperative pathological examination present both pathological change of synovial chondromatosis and synovial tuberculosis (Captured by Mshot microscope digital camera MD50 with Logene PathQC imaging software, Fig. 1e), SC forms loose body in knee joint and synovial tuberculosis shows a tubercular granuloma with Langerhans giant cells and hyperplasia of epithelioid cells (Fig. 1d, e). Along with Tuberculosis DNA (TB-DNA) test (+) confirms the coexistence of synovial chondromatosis combined with synovial tuberculosis of knee joint. After surgery the patient was told to receive anti-tuberculosis treatment for 1 year and long-leg splint for 6 weeks. After 6 months follow-ups, knee swelling and claudication get totally recovered and the gait of patient recover back to normal.

## Discussions and conclusions

The knee joint tuberculosis of this case starts as extrapulmonary tuberculosis. The clinical manifestations of extrapulmonary tuberculosis are complex and there is no unified diagnostic standard, so it is difficult to diagnose and cause delay in treatment. Generally speaking the suspected patient’s history should be inquired carefully, and detailed clinical evaluation should be carried out to find the typical symptoms and signs of the corresponding system and parts, such as low fever, night sweats, ineffective anti infection treatment, etc. Above all, ESR, PPD, chest X-ray and other examinations should be carried out, and whether there is a history of tuberculosis contact should be inquired to assist comfirm the diagnosis. Knee joint tuberculosis always appears asymptomatic, no local swelling and dysfunction. Large lesions can lead to local discomfort or pain, most cases get symmetrical onset, more commonly seen on the medial and posterior side of the distal femoral metaphysis, the tibia or the upper end of the fibula, and often have bilateral symmetry as characteristic. However, our case has unilateral knee joint local swelling and dysfunction, combined with the predilection site and clinical manifestations, early diagnostic biopsy of the knee is the key to the diagnosis and treatment of the disease; it can also be used for TB-DNA detection.

The onset time in this child was 3 years old. In this case, the intra-articular loose body was composed of cartilage, which is a solid change without obvious calcification. No loose body was seen on X-ray, so it is easy to be misdiagnosed as other diseases. Finally, the patient was suspected as SC by MRI with a large number of loose bodies. We used open synovectomy and loose body resection of the knee joint, combined with postoperative pathological findings diagnosed as osteochondromatosis of the knee joint synovial, and TB-DNA test comfirmed tuberculosis of the knee joint synovial.

The incidence rate of this disease is very low in adults, and is even less frequent in children. Therefore, the literature is basically in the form of case reports. The minimum age of synovial chondromatosis reported at present is 2 years old [5], but there are few reports of SC complications Ben Efrima reported a 37 year old female hip joint SC with pigmented villonodular synovitis [6], Jones reported 2 cases of SC with peripheral nerve compression [7], Monestier reported 2 cases of foot fat deposition combined with SC [8]. However, no case of knee synovial chondromatosis combined with tuberculosis has been reported in the literature.

In summary, it is a rare case of synovial chondromatosis combine with tuberculosis in the knee joint, according to pathologic change and literature, we speculate this association is coincidental. This is the first case of knee synovial chondromatosis combined with tuberculosis in the literature. Careful investigation of children with knee pain is recommended to avoid misdiagnosis, SC combine with tuberculosis should be considered a differential diagnosis in a child with knee pain.

## Data Availability

The datasets used and/or analysed during the current study are available from the corresponding author on reasonable request.

## Abbreviations

SC: Synovial chondromatosis
MRI: Magnetic Resonance Imaging
CT: Computerized tomography
TB: Tuberculosis
ESR: Erythrocyte Sedimentation Rate
CRP: C-reactive protein
